# Characterizing Environmental Background Microflora and Assessing Their Influence on *Listeria* Persistence in Dairy Processing Environment

**DOI:** 10.3390/foods14101694

**Published:** 2025-05-10

**Authors:** Vaishali Poswal, Sanjeev Anand, Brian Kraus

**Affiliations:** 1 Department of Dairy and Food Science, South Dakota State University, Brookings, SD 57006, USA; vaishali.poswal@jacks.sdstate.edu; 2 Wells Enterprises Inc., Le Mars, IA 51031, USA; bnkraus@bluebunny.com

**Keywords:** *Listeria monocytogenes*, food processing environment, biofilm, floor swab, air samples

## Abstract

*Listeria monocytogenes* (*Lm*) may persist in food processing environments (FPEs) alongside diverse background microflora. While microbial communities in FPEs can influence *Lm* survival, their role in supporting or suppressing its growth remains unclear. This study aimed to characterize the microflora in floor swabs and air samples collected from a dairy processing facility across three seasons and assess their potential impact on the growth of a *Lm* test strain previously isolated from a dairy processing environment. A total of 167 environmental isolates, representing 30 bacterial genera, were identified. *Pseudomonas* was consistently prevalent across all sample types. Seasonal shifts in bacterial genera were observed, with differences in microbial composition and relative abundance between production lines with and without *Listeria innocua* occurrence. Microflora distribution appeared more influenced by environmental and operational factors than by spatial proximity. Co-culture growth assays revealed no competitive exclusion of the *Lm* test strain, and no zones of inhibition were observed in antimicrobial assays using cell-free extract and dialyzed cell-free extract from environmental isolates against *Lm*. These findings suggest that *Lm* could potentially establish itself within mixed microbial communities in dairy processing environments, emphasizing the complexity of microbial interactions in FPEs and their potential role in *Lm* persistence.

## 1. Introduction

*Listeria monocytogenes* poses a significant threat to public health, leading to illnesses that, in severe cases, may necessitate hospitalization or even result in death. Thus, it is crucial to prevent the contamination of food and food production environments (FPEs) by this pathogen. Despite continuous efforts of the food industries to eliminate pathogens, *Listeria monocytogenes* (*Lm*), a causative agent of listeriosis, remains persistent in FPEs [1,2,3] and has a high fatality rate [4]. *Lm* has been implicated in numerous foodborne outbreaks linked to a wide array of ready-to-eat food products, including dairy (milk, cheese, ice cream), seafood, vegetables, and processed meats [5]. For instance, in 2024, a multistate *Listeria* outbreak linked to meats resulted in multiple hospitalizations and deaths in the United States [6]. *Lm* is a persistent pathogen in food processing environments (FPEs), where it can survive for years or even decades due to its high-stress tolerance and ability to form resilient biofilms on food contact surfaces [7,8]. Persistent strains of *Lm* are defined as genetically similar isolates that are repeatedly recovered from the same location over extended periods, often six months or more. These isolates are typically indistinguishable in molecular profiles, as determined by genome-based subtyping methods, suggesting long-term adaptation to specific environmental niches [9,10]. However, it is important to note that not all *Lm* strains exhibit persistence; some are recovered only sporadically and do not show evidence of long-term establishment [11]. In FPEs, *Listeria* has been frequently isolated from floors, drains, standing water, and equipment, with floor drains acting as potential hotspots due to their difficult cleaning [3,12,13,14,15]. *Lm* activates diverse stress tolerance mechanisms in response to environmental stresses, including the general stress response regulator SigB, heat shock protein GrpE, cold shock protein D, oxidoreductase, and glutathione reductase, further enhancing its persistence in these environments [4,16].

In food processing environments (FPEs), *Lm* is often found alongside background microflora, leading to interactions that can influence its behavior and persistence [17,18]. Within multispecies biofilms, these interactions may be competitive, where *Lm* growth is suppressed by other microorganisms, or cooperative, resulting in enhanced proliferation and survival; in some cases, interactions may be neutral [18]. Previous studies have demonstrated that in mixed biofilms, such as those involving *Flavobacterium* spp., *Lm* exhibits increased surface attachment, prolonged survival, and greater resistance to sanitizers compared to single-species biofilms [19]. Similarly, co-cultivation with *Pseudomonas* spp. has been shown to enhance biofilm production relative to *Lm* monocultures [18,20]. Nevertheless, other reports indicate that *Lm* cell densities can decline in mixed-culture biofilms due to nutrient competition, although certain strains may adapt by strengthening their attachment mechanisms [2]. Additionally, associations with specific species, such as *Kocuria varians*, have been found to facilitate the easier detachment and removal of *Lm* during cleaning processes [2]. While multispecies communities may offer *Lm* protection from environmental stress, their overall contribution to long-term persistence remains poorly understood and requires further investigation. Beyond biofilm-specific interactions, microbial communities in FPEs engage in broader ecological processes such as quorum sensing, competitive exclusion, and the production of antimicrobial compounds that can inhibit competing microflora [21,22]. For example, *Bacillus subtilis* isolated from Korean fermented soybean paste has been shown to produce antimicrobial substances effective against *Lm* strains [23]. A deeper understanding of these microbial interactions is essential for developing more effective strategies to control *Lm* within food processing environments.

Previous studies have characterized the background microflora in FPEs [15,24], with some examining their effect on *Lm* growth using samples collected over relatively short durations [21,25]. Unlike these studies, our research investigates microbial communities in a large-scale dairy production system across three seasons, providing a more comprehensive analysis. Understanding microflora composition is essential to determine whether background microflora supports or limits *Lm* growth. This study analyzes microbial communities in air and floor swab samples and examines their interactions with a *Lm* test strain previously isolated from the dairy processing environment. These findings enhance the understanding of microbial dynamics in FPEs and their role in *Lm* persistence.

## 2. Materials and Methods

### 2.1. Sample Collection from a Dairy Processing Plant

**Figure 1:**
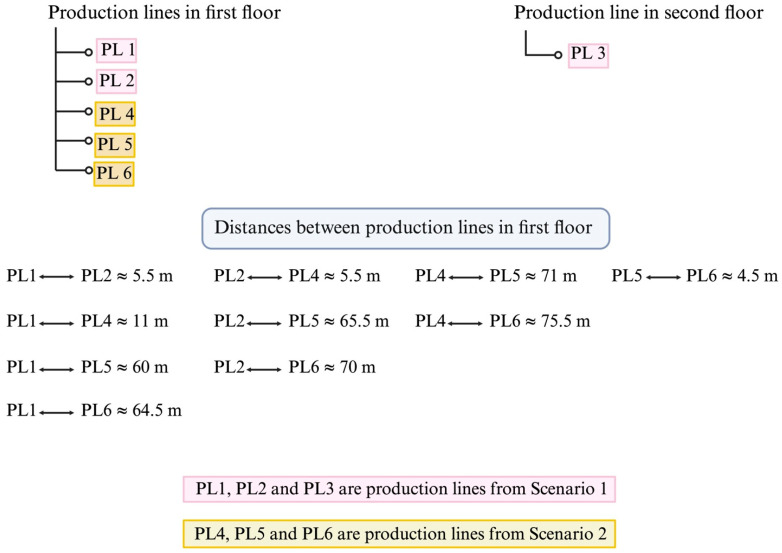
Layout and distance (meters) between the sampled sites located on the first floor and second floor of the processing area.

Environmental samples were collected from a commercial dairy plant with fully automated production processes located in the Midwest region of the United States in the fall (December 2023), spring (March 2024), and summer seasons (June 2024). Environmental samples comprising air and floor swab samples were collected simultaneously from six production lines named PL1, PL2, PL3, PL4, PL5, and PL6, manufacturing different varieties of ice cream. Samples collected from production lines were categorized into two scenarios: Scenario 1 included three production lines (PL1, PL2, PL3) showing seasonal occurrences of *Listeria* species, primarily *L. innocua*, as confirmed by industrial scale ribotyping, though none recorded *L. monocytogenes*. Scenario 2 included three production lines (PL4, PL5, PL6) with rare or no recorded *Listeria* species isolation. In total, six production lines were sampled for air and floor microflora across three distinct seasons. Sampling was performed only once per season for all production lines. Since *Lm* was not identified in any of the samples, a previously isolated strain was used throughout this study. This test isolate was originally isolated in May 2019 from a Zone 3 (non-food contact) surface in a dairy processing facility. The isolate had been previously characterized by ribotyping and whole-genome sequencing. Multilocus sequence typing analysis had been previously performed in our laboratory by uploading the assembled FASTA file to the BIGSdb-Pasteur database (ID: 101844), which assigned the strain to clonal complex CC5, sublineage SL5, sequence type ST5, and phylogenetic lineage I [26]. Sampling was conducted during processing hours at various locations along the production lines, including areas beneath the freezer, at the filling head, near the flavor tank, around drains, and near the ingredient feeder. The distances between the production lines were measured to study any possible relationship with the occurrence of environmental microflora based on their spatial proximity (Figure 1).

#### 2.1.1. Collection of Floor Swab Samples

Floor swab samples were collected from five adjacent locations at each production line using EZ Reach^TM^ sponge samplers (World Bioproducts©, Bothell, WA, USA) pre-moistened with HiCap neutralizing broth (World Bioproducts©, Bothell, WA, USA). Each sample covered a 10 × 10 cm^2^ area on the floor [27]. The five sponges from each site were placed in sterile bags, stored in a cooler during transport, and processed within 24 h.

#### 2.1.2. Collection of Air Samples

Airborne cultivable microorganisms were sampled using a Biotest HYCON Air Sampler (RCS, SN 27617) (flow rate: 40 L/min) for 8 min with a tryptic soy agar (TSA) in flexible foil (HYCON^®^, Merck KGaA, Darmstadt, Germany) [28]. Sampling was performed at a height of 1.0 m above the floor, and HYCON^®^ Agar Strips TC (Total Count, 1442530050) was used to determine the total cultivable microorganisms, following the manufacturer’s guidelines. Samples were collected from each production line at four distinct locations positioned in the north, south, east, and west directions relative to the processing line. The strips were transported to the laboratory under cool conditions by overnight shipping.

### 2.2. Isolation and Identification of Microflora from Environmental Samples

#### 2.2.1. Isolation and Identification of Floor Swab Microflora

The floor swab samples were cultured on TSA. The sponge swabs were squeezed to collect the samples in a 50 mL centrifuge tube (Fisher Scientific, Waltham, MA, USA). Further dilutions up to the 5th were prepared using Phosphate Buffer Saline (PBS) solution (pH 7.2) (Fisher Scientific, Waltham, MA, USA), with the 3rd to 5th dilutions spread-plated in triplicate onto pre-poured TSA plates and incubated at 37 °C for 24–48 h. The counts were determined as log_10_ colony-forming units per square centimeter of the area swabbed. Colonies with distinct morphologies were Gram-stained and streaked individually onto TSA plates, with each streaked colony treated as a separate isolate. Single colonies from each isolate were then identified using Matrix-Assisted Laser Desorption/Ionization-Time of Flight (MALDI-TOF) at the Diagnostic Lab, Veterinary and Biomedical Sciences Department, South Dakota State University [29]. After identification, the percentage abundance of each genus was calculated by grouping colonies on the selected dilution of TSA agar with similar morphology to the identified isolates. The combined counts of these grouped colonies were then divided by the total number of colonies observed on the TSA agar to determine the percentage abundance of each genus.

#### 2.2.2. Isolation and Identification of Air Microflora

The agar strips were incubated at 37 °C for 24–48 h to allow visible colony formation. The colonies on each strip were counted, and the colony-forming units per cubic meter of air (CFU/m^3^) were determined by dividing the colony counts by the total volume of air sampled. For each processing line, the mean log_10_ CFU/m^3^ was calculated using counts from four samples collected in the north, south, east, and west directions. Distinct colonies with varied morphologies on the aerobic plate count (APC) strips were Gram-stained and individually streaked onto TSA plates, with each streaked colony treated as a separate isolate. Single colonies from each isolate were then selected for identification using Matrix-Assisted Laser Desorption/Ionization-Time of Flight (MALDI-TOF) at the Diagnostic Lab, Veterinary and Biomedical Sciences Department, South Dakota State University [29]. After identification, the percentage abundance of each genus was determined. Colonies on the agar strips exhibiting similar morphology to the identified isolates were grouped, and their combined counts were divided by the total number of colonies observed on the strips to calculate the percentage abundance of each genus.

For glycerol stock preparation, isolates from air and floor swab samples were subcultured in tryptic soy broth (TSB) at 37 °C for 24–48 h and centrifuged at 3500 rpm for 20 min. The resulting pellets were washed twice with PBS and resuspended in PBS. The suspensions were transferred to 1.8 mL cryogenic vials containing sterile beads and glycerol and stored at −80 °C until further use [30].

### 2.3. Co-Culture Growth of Environmental Microflora with the L. monocytogenes (Lm) Test Strain

#### 2.3.1. Multispecies Co-Growth Study

The *Listeria monocytogenes* (*Lm*) test strain previously isolated from a dairy processing environment was used throughout this study. To evaluate direct co-exclusion of *Lm* using floor swab samples, 0.1 mL of swab sample was inoculated with 2 log_10_CFU/mL of *Lm* in 10 mL of Tryptic Soy Broth (TSB). The mixture was incubated at 37 °C for 24 h. Following incubation, the samples were plated on MOX agar in triplicate to enumerate *Lm*.

In a separate experiment, dominant bacterial species from the floor swab samples were identified using MALDI-TOF analysis. Prominent species from each genus for each sample were combined at 2 log_10_CFU/mL for each species and mixed with 2 log_10_CFU/mL of *Lm* in 10 mL of TSB. The mixtures were incubated at 37 °C for 24 h. After incubation, the samples were plated on MOX agar to enumerate *Lm*.

#### 2.3.2. Single-Species Co-Growth Study

All the isolates were individually studied for co-culture growth along with the *Lm* test strain. The overnight-grown individual isolates and *Lm* were added in a 1:1 ratio, 100 µL of *Lm* and 100 µL of each isolate, 2 log_10_CFU/mL each, in 10 mL Brain Heart Infusion (BHI) broth. They were incubated at 37 °C for 24 h. At 24 h, total plate counts were taken on TSA for co-culture counts and modified Oxford agar (MOX) for *Lm* counts.

### 2.4. Screening Isolates for Their Potential Inhibition of the L. monocytogenes (Lm) Test Strain

#### 2.4.1. Using the Cell-Free Extract of Individual Environmental Isolates

Isolates grown individually for 24 h were centrifuged at 3500 rpm for 20 min and micro-filtered through a 0.22 µm Millipore filter (Stericup Quick Release, Merck Millipore, Burlington, MA, USA) to obtain cell-free extract (CFE) [31]. A 500 µL aliquot of 24 h grown *Lm* (10^7^–10^8^ CFU/mL) and 72 h grown *Micrococcus luteus* (control) [32] was added to separate 5 mL portions of 0.75% soft agar and overlaid on TSA plates. For the spot-on lawn method, 3 µL of CFE was spotted on the TSA plates with *Lm* and *Micrococcus luteus* (control) in soft agar [33]. For the well assay method, wells were made with a 6 mm diameter borer, and 20 µL of CFE was pipetted in each well [31]. To allow CFE to diffuse in the agar, the plates were kept at 4–8 °C for 30 min and then incubated at 37 °C [33]. Bacterial lawns were monitored at every 6 h interval until 24 h for the formation of any zone of inhibition, an indicator of antimicrobial activity.

#### 2.4.2. Analyzing the Antimicrobial Activity of the Dialyzed Cell-Free Extracts of Individual Isolates

The CFE of isolates was dialyzed individually using SnakeSkin^TM^ Dialysis Tubing with a 3.5K MWCO, 22 mm I.D. (Fisher Scientific, Waltham, MA, USA). Freshly prepared CFE was pipetted into the dialysis tubing, secured with SnakeSkin^TM^ Clips, and concentrated using G-Biosciences powder (Geno Technology Inc., St. Louis, MO, USA) to reduce the solution volume through water absorption [34]. The dialysis tube, containing the sample, was stored at 4–8 °C, with frequent inspections to replace the wet concentrator powder as needed until the desired volume was achieved. Once the target volume was reached, the concentrator powder was removed, and the antimicrobial activity of the dialyzed CFE was evaluated using the previously described spot-on-lawn and well assay methods.

### 2.5. Screening of Floor Swab Samples for Their Potential Inhibition of the L. monocytogenes (Lm) Test Strain

Antimicrobial activity involves the ability of the floor swab samples to inhibit the growth of *Lm* and *M. luteus* (control) and was assessed using the floor swab samples micro-filtered through a 0.22 µm Millipore filter. Evaluation was performed using the spot-on-lawn and well-diffusion assay methods, as described earlier in this article.

### 2.6. Statistical Analysis

Each experiment was conducted with three replicates for every analysis. Data analysis was performed using Origin Pro software (Version 2024b (10.15) (Northampton, MA, USA). For standard plate count data, data analysis was conducted using three-way ANOVA, where scenario, season, and production lines were compared as separate treatments. The Tukey test with 95% confidence limits was used for comparing means. Also, the graphs were plotted using Microsoft Excel (Version 16.87 (24071426)).

## 3. Results and Discussion

### 3.1. Characterization and Comparison of the Microflora from Floor Swabs and Air Samples

The standard plate count (SPC) for floor swab samples ranged from 3.78 to 7.09 log_10_ CFU/cm^2^, while air samples exhibited a range from 1.51 to 1.98 log_10_ CFU/m^3^ (Table 1). The microbial counts from the air samples were lower as compared to those reported in a previous study conducted in commercial dairy plants (1.84–2.93 log_10_ CFU/m^3^) [27]. The floor swab count was comparable to that found in fresh-cut produce processing plants (5.83 log_10_ CFU/cm^2^) [35].

A total of 167 isolates were identified from all samples, comprising 30 bacterial genera, 2 mold genera (*Aspergillus* and *Penicillium*), and yeasts. The air samples exhibited greater genera diversity compared to the swab samples. Five bacterial genera—*Enterobacter*, *Enterococcus*, *Klebsiella*, *Pantoea*, and *Pseudomonas*—were common to both air and swab samples. *Bacillus* and *Micrococcus* species were exclusively isolated from air samples. *Bacillus*, commonly associated with soil and plants, is likely introduced into the processing environment through outdoor air, ventilation systems, and human clothing and footwear [27]. A higher prevalence of *Bacillus* and *Micrococcus* in air samples compared to surface samples has also been observed in dairy processing facilities [27]. Lactic acid bacteria, namely, *Leuconostoc* spp., were found in the swab sample, and *Enterococcus* spp., in the air sample. The lactic acid bacteria can act against spoilage organisms and are frequently isolated from dairy processing environments [24]. Overall, in the floor swab samples, the three most abundant species in the floor swab samples were *Klebsiella oxytoca*, *Shewanella putrefaciens*, and *Serratia liquefaciens*. Similarly, in the air samples, the most abundant species were *Micrococcus luteus*, *Staphylococcus pasteuri*, and *Pseudomonas fulva*. The twenty most predominant species isolated from both floor swabs and air samples are given in Table 2. In this study, *Lm* was not detected at any processing sites in floor swabs or air samples across all seasons.

*Pseudomonas* was the only genus consistently present across all sample types, with the highest prevalence in swab samples and the fourth highest in air samples. This is unsurprising, as *Pseudomonas* is one of the most reported genera found post-sanitation across various FPEs. *Pseudomonas* spp. are associated with diverse environmental niches, likely due to their ability to withstand stress conditions such as low temperatures, nutrient scarcity, sanitizers, and shear force, and their notable capability to utilize quaternary ammonium compounds as an energy source [36,37]. *Pseudomonas* has been isolated from various FPEs, including ice cream production [38,39], wooden vats for cheese [40], cheese ripening rooms [21], ultrafiltration and reverse osmosis membranes [41], equipment surfaces [24,42], and floor drains [15]. Their prevalence in the FPEs can be attributed to their ability to produce extracellular DNA and exopolysaccharides, which facilitate biofilm formation. Moreover, *Lm*, if present, may enhance matrix production in biofilms with *Pseudomonas*, which can protect both species from desiccation and disinfection [36].

The observed differences in the genera identified between air and swab samples suggest that aerosolization from the floor and drains within the processing area may be limited. The findings indicate a higher diversity of microbial genera in air samples compared to swab samples. This disparity may be attributed to the routine cleaning of floors and drains following processing runs, which likely reduces the microbial load on surfaces. A study conducted in dairy processing environments observed higher airborne microbial concentrations in traditional dairies compared to commercial ones, highlighting the influence of environmental factors on bioaerosol concentration and diversity [27]. Various factors, including processed materials, manufacturing practices, high-pressure spraying, human activity, air circulation, and microclimate conditions (temperature and relative humidity), play a crucial role in shaping microbial loads in the air of food processing plants [27,43,44].

The air and floor swab sampling in this study was performed three hours into production. However, the composition of the microflora in food processing environments may vary significantly after extended production periods (e.g., 24 h or more), depending on the ability of resident microorganisms to develop biofilms. This temporal variation in microflora may also influence the attachment and biofilm formation of *Lm* if present as environmental contaminants. Cleaning and sanitation practices, including their frequency and effectiveness, also play a major role in shaping the abundance and distribution of microbial communities in dairy and food processing environments [1]. Additionally, factors such as surface characteristics, ecological interactions, nutrient availability, and cleaning protocols contribute to the variability in resident microflora composition within a facility and across different processing plants [24]. In this study, TSA was used as the growth medium to cultivate the background microflora from the floor samples. Consequently, this study was limited to culturable microorganisms and their potential influence on the growth and survival of *Lm* as previously also reported in some studies [45].

#### 3.1.1. Seasonal Variation of Microflora

Seasonal comparisons revealed significant differences in the SPC of floor swabs and air samples (Table 1). These findings suggest that seasonal factors may influence microbial load on floor surfaces and in the air of FPEs. The microbial load was highest in summer for floor samples from four processing lines and air samples from three processing lines, followed by fall and spring for both sample types. The seasonal variation in microbial load for these processing lines aligns with local temperature fluctuations, with summer having the highest temperatures, followed by fall and spring. This trend suggests that elevated temperatures promote microbial proliferation in food processing environments (FPEs), a phenomenon well-understood in other food industries, such as meat and seafood processing [46].

**Figure 2:**
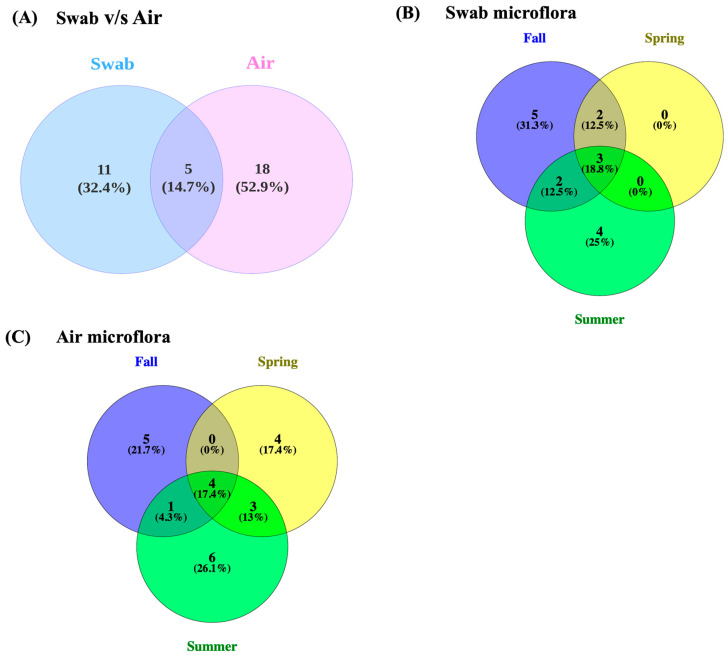
Venn diagram of genera distribution in (**A**) swab and air samples, (**B**) swab microflora among different seasons, and (**C**) air microflora among different seasons.

The composition of bacterial genera also varied seasonally in both sample types. The genera present were compared in different seasons for both sample types (Figure 2). Out of the total of 16 genera found in the swab microflora, including yeast, 9 were only found in one of the three seasons, and out of a total of 23 genera, including yeast and molds found in the air, 15 were found only in one of the three seasons. This indicates a high variation in the microbial composition of the environment of dairy processing plants during different seasons. Floor swab samples for each production line were collected from five adjacent locations, ensuring spatial coverage within each line. This sampling approach provides valuable preliminary data on seasonal variation in bacterial communities. Multi-year studies with increased sampling frequency are needed to better assess seasonal trends in the dairy processing environment.

**Figure 3:**
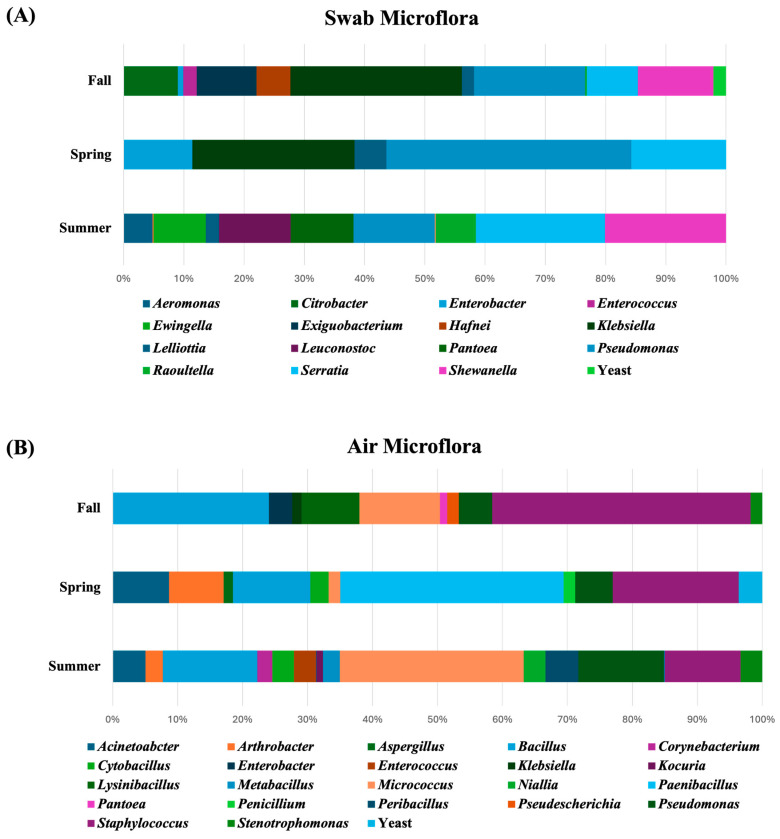
Relative abundance of the bacterial, yeast, and mold genera in (**A**) swab and (**B**) air samples among different seasons.

The highest microbial diversity was observed in the air samples collected during the summer, followed by the swab samples from the fall season. The most abundant genera in the swab samples were *Klebsiella* in the fall, *Pseudomonas* in the spring, and *Serratia* in the summer. In contrast, the air samples were dominated by *Staphylococcus* in the fall, *Paenibacillus* in the spring, and *Micrococcus* in the summer. Figure 3 shows the relative abundance of the microflora in floor swabs and air samples across the different seasons throughout the year. The observed shifts in genera align with findings from previous studies in fresh produce processing facilities [47,48] and dairy processing environments [49], which reported seasonal changes in the environmental microbiomes of food processing facilities.

#### 3.1.2. Scenario 1 and 2: Distribution of Microflora

**Figure 4:**
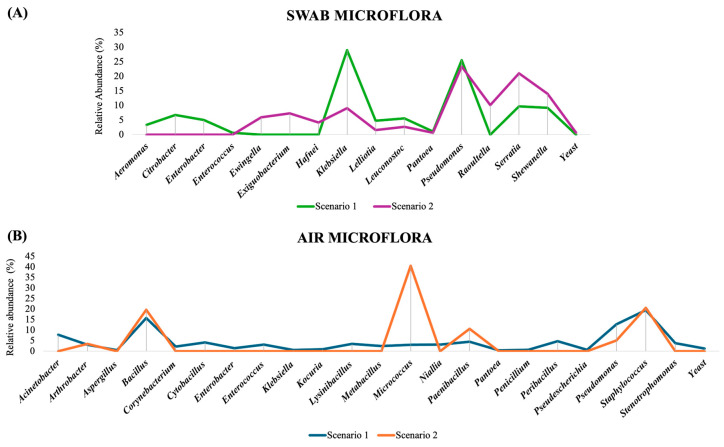
Comparative distribution of microbial genera in two different scenarios in (**A**) swab microflora and (**B**) air microflora (Scenario 1 included sites with seasonal occurrences of *Listeria* species, primarily *L. innocua*, and Scenario 2 included sites with some or no recorded *Listeria* species isolation).

The standard plate counts (SPCs) varied significantly between the two sampling scenarios for air and floor swab samples, indicating differences in the microbial load. Significant differences in environmental microflora and their relative abundance were observed between the two scenarios. Comparing Scenario 1 (including sites with seasonal occurrences of *Listeria* species, primarily *L. innocua*) and Scenario 2 (including sites with some or no recorded *Listeria* species isolation) in swab samples, *Klebsiella*, the most abundant genus in Scenario 1 comprised approximately 29% of the microbial population and was estimated to be about 9% in Scenario 2 (Figure 4). On the other hand, *Pseudomonas*, the second most abundant genus in Scenario 1, estimated to be about 25%, was the most abundant genus in Scenario 2 with a comparable value of 23%. In a similar study conducted in an ice cream manufacturing environment, *Pseudomonas* was the most abundant genus in two of the three facilities sampled, while *Klebsiella* was detected in only one facility [39].

Distinct genera were detected in swabs and air samples across two scenarios. In Scenario 1, genera like *Aeromonas* and *Citrobacter* were found in swab samples, while genera including *Acinetobacter*, *Aspergillus*, and *Corynebacterium* were found in air samples; in Scenario 2, additional swab genera were observed, but the air sample contained all the genera present in Scenario 1 (Table 3). The significant differences in environmental microflora observed between the two scenarios align with previous findings, where variations in microbial composition were associated with differences in *Lm* occurrence [21,50]. A prior study reported that facilities with higher *Lm* prevalence exhibited distinct environmental microbiota compared to other facilities with lower *Listeria* presence. Notably, *Pseudomonadaceae* predominated in environments with *Listeria*, reinforcing its role as a biofilm former that can enhance *Listeria* survival under cleaning and sanitation conditions [50]. *Klebsiella* was also found to be the dominant microbiota in the *Listeria*-positive floor drains [51,52]. In a similar study conducted in food production facility drains, differences in microflora were observed between *Listeria*-positive and *Listeria*-negative sites. One study reported an increased presence of the genera *Prevotella* and *Janthinobacterium* in *Listeria*-negative drains, while *Enterococcus* and *Rhodococcus* were more abundant in *Listeria*-positive drains [21]. Comparing Scenario 1 and Scenario 2 in air samples, only 6 genera were present in the Scenario 2 samples, of which *Micrococcus* was most abundant (40.5%), on the other hand, 23 genera including 20 bacterial genera, 2 mold genera, and yeast were found in Scenario 1 of which *Staphylococcus* (20.5%) and *Bacillus* (19.5%) were more prevalent. Significant differences in air microflora composition and relative abundance were observed between the two scenarios, likely influenced by variations in product types manufactured on the production lines and differences in human traffic patterns.

#### 3.1.3. Distribution of Microflora Across Production Lines (PL)

The SPC for both air and floor swab samples varied significantly across the production lines, suggesting that localized factors, such as equipment layout, traffic patterns, or airflow, may contribute to the distribution of microbial contamination. Similarities in the microbial composition of floor swab samples were observed across different production lines. For PL1 and PL2 (located 5.5 m apart, Figure 1), the dominant genera included *Lelliottia* and *Pseudomonas* in the fall, *Pseudomonas* in the spring, and *Serratia* in the summer for floor swab samples. For PL2 and PL4 (also 5.5 m apart), *Pseudomonas* was prevalent in the fall, *Pseudomonas* and *Serratia* in the spring, and *Leuconostoc* in the summer. Additionally, PL1 and PL4 (separated by 11 m) shared *Pseudomonas* in the spring and *Lelliottia* and *Pseudomonas* in the fall. For sites PL 5 and PL 6 (4.5 m apart), *Pseudomonas* was dominant in the spring, while *Shewanella* was prevalent in the summer. Their shared environmental conditions due to proximity may explain the similarity in the microflora observed across these processing sites. Interestingly, despite the spatial separation of PL3 from other sites, it shared similar microbial genera with them for floor swab samples, suggesting that the distribution of floor swab microflora was not strictly influenced by site proximity. This observation implies that microbial dispersal within the plant might be influenced by factors beyond physical distance, such as air circulation, personnel movement, surface type, cross-contamination from equipment and workers, or equipment sharing [53]. The compositions of bacterial communities in biofilms residing on high-nutrient surfaces have been reported to differ significantly from those on low-nutrient surfaces [54]. Moreover, previous studies indicate that persisting organisms are limited by selection due to routine sanitization [54]. As all PLs are sanitized regularly and manufacture the same type of product, only persistent organisms can potentially survive, making the similarity in microflora justifiable.

The dominant genera identified in the air samples included *Bacillus*, *Staphylococcus*, *Micrococcus*, and *Pseudomonas*, irrespective of the processing line. There was no apparent trend in the microbial composition of air samples based on the distances between the production lines, while some genera were consistently present across all air sample locations. This non-uniformity could be attributed to factors such as airflow patterns, ventilation systems, or shared environmental conditions [44]. These findings provide a foundation for future large-scale investigations across multiple processing facilities, allowing comparisons of microbial compositions in different production lines and distant locations. In addition, a multi-year study could offer deeper insights into long-term microbial trends.

### 3.2. Co-Culture Growth of Environmental Microflora with L. monocytogenes (Lm) Test Strain

The multispecies co-growth study, performed to evaluate the ability of floor swab sample microflora to inhibit the growth of *Lm*, revealed no suppression of *Lm* growth. After 24 h of incubation, the *Lm* counts ranged from 4.35 to 5.5 log_10_CFU/mL, indicating no inhibitory effect of the environmental background microflora from any of the tested swab samples on *Lm*. Similarly, when the dominant species identified from each swab sample were co-cultured with *Lm* in TSB, the *Lm* counts ranged from 2.35 to 3.53 log_10_CFU/mL after 24 h of incubation. These experiments, which were also performed separately for each swab sample, showed no evidence of suppression of *Lm* growth by the dominant species identified from the air or swab samples.

Further investigation through co-culture studies with individual isolates was performed to determine whether any specific isolate could limit the growth of the *Lm* test strain. After 24 h of incubation, total plate counts for the mixed cultures ranged from 4.60 to 6.47 log_10_ CFU/mL. Meanwhile, the *Lm* test strain counts on MOX agar ranged from 2.48 to 3.78 log_10_CFU/mL. None of the tested isolates exhibited the ability to suppress or inhibit the growth of the *Lm* test strain. Interestingly, the *Lm* test strain population consistently exceeded that of the co-cultured isolates, suggesting that the *Lm* test strain outcompeted the other microorganisms in the co-growth system. This competitive advantage may highlight the resilience of *Lm* in mixed microbial environments, particularly in the dairy processing environment.

Previous research suggests that non-pathogenic microflora may sometimes limit or suppress the growth of pathogenic bacteria, including *Lm*, either within multispecies biofilms or as individual strains [55]. However, some other studies have reported that the presence of competing microflora can, in some cases, facilitate the establishment of *Lm* biofilm [2,19]. For instance, a co-culture study involving *Lm* and other bacterial species such as *Escherichia coli*, *Pseudomonas fluorescens*, and *Lactobacillus plantarum* found that *Lm* was suppressed when introduced at a lower concentration relative to the competitor. In contrast, when *Lm* was introduced at a higher concentration, it outcompeted the other species and dominated the co-culture [56]. In the present study, none of the environmental microflora from floor swab samples exhibited an inhibitory effect on the *Lm* test strain regardless of their initial abundance. Instead, the *Lm* test strain consistently outgrew the other microbial species in co-culture conditions. These findings indicate that *Lm* could potentially establish itself in mixed microbial communities, particularly when favorable conditions support its growth. However, it is important to note that the relative abundance of *Lm* compared to the background microflora may vary under natural conditions, which could influence the dynamics observed in this study. Moreover, this hypothesis would need to be further validated through single and mixed species biofilm development along with the *Lm* test strain, as in a natural environment, interactions among multiple bacterial species may play a different role in inhibiting or limiting the growth of *Lm* [57,58]. Expanding this research to mixed species biofilms could enhance the understanding of these microbial dynamics and improve the environmental cross-contamination control strategies. At this point, the results from this study may be helpful in developing targeted cleaning and risk management models aimed at minimizing the persistence of *Lm* in food processing environments.

### 3.3. Screening Isolates for Their Potential Inhibition of L. monocytogenes (Lm) Test Strain

The antimicrobial activity of the individual isolates was evaluated using both the spot-on-lawn and well-diffusion assays against *Lm* and *M. luteus* (control). None of the isolates exhibited any zones of inhibition against *Lm* and *M. luteus* using the CFEs of environmental microflora. Even after concentrating the CFEs by dialysis to get the dialyzed cell-free extract, there were no zones of inhibition for any of the isolates. The protein concentration in the dialyzed CFE was approximately 55–75 mg/mL for all the isolates. Thus, under the test conditions and at the concentration of protein obtained by CFE dialysis, none of the isolates exhibited antimicrobial activity. Additionally, the antimicrobial activity of the CFE from direct swab samples was tested against *Lm* and *M. luteus*, with no zones of inhibition observed, indicating similar results. On the other hand, a previous study conducted on the isolates from small cheese-producing farms demonstrated that some of the isolates gave a zone of inhibition of a maximum of 2–3 mm, indicating a weak inhibitory effect against *Lm* [25]. The antimicrobial effects of microorganisms are influenced by various factors, including the specific strain, environmental stress conditions, temperature, and inoculum level [59,60,61]. The assessment of potential antimicrobial activity involved incubating swab samples in TSB for 24 h at 37 °C. While TSB supports the growth of a broad spectrum of bacteria [62], variations in microbial metabolic activity under different environmental conditions and growth media compositions could influence the extent of antimicrobial interactions observed. Moreover, it would be useful to conduct studies involving single and mixed species biofilms to evaluate the *Listeria* persistence in the presence of environmental microflora.

## 4. Conclusions

This study explored the influence of environmental background microflora on the growth and persistence of *Listeria*. Significant seasonal variations in microbial composition were observed, highlighting the dynamic nature of microbial communities in these environments. The distribution of microflora across production lines was shaped more by environmental conditions than by spatial proximity, reflecting the influence of operational factors on microbial presence. Despite identifying a diverse range of microbial genera, including *Pseudomonas*, *Klebsiella*, *Micrococcus*, and *Staphylococcus* from floor swabs and air samples, no evidence of competitive exclusion or inhibitory effects against the *Lm* test strain was observed in co-culture growth assays. Similarly, evaluation of the antimicrobial activity of the cell-free extracts and the dialyzed cell-free extracts from the environmental isolates showed no inhibition against the *Lm* test strain under the test conditions and at the protein concentration obtained by dialysis. These findings thus indicate that, under the tested conditions, the *Lm* test strain can persist and grow in the presence of various Gram-positive and Gram-negative background microflora. Given that microbial interactions in biofilms may differ from those in planktonic cultures, further investigation is recommended to assess the role of background microflora in *Lm* persistence within biofilms. In view of this, single and multispecies biofilm studies are being conducted in our lab to examine the persistence of *Lm* within mixed microbial communities and interactions among species that may impact its survival. Expanding this research to mixed-species biofilms is likely to provide a more comprehensive understanding of microbial dynamics and develop environmental cross-contamination control strategies.

The ability of *Lm* to survive alongside diverse microbial communities underscores the need for rigorous cleaning and sanitation protocols in dairy processing facilities. A comprehensive understanding of these microbial dynamics would aid in developing targeted approaches to mitigate the persistence of *Lm* in dairy processing environments and enhance food safety management practices.

## Figures and Tables

**Table 1:** Standard plate counts of floor swab samples (log_10_ CFU/cm^2^) and air samples (log_10_ CFU/m^3^) collected from the dairy processing plant during various seasons.

Scenario	PL ^1^	Floor Swab Sample (log_10_ CFU/cm^2^)			Air Samples (log_10_ CFU/m^3^)		
		Fall	Spring	Summer	Fall	Spring	Summer
Scenario 1	PL1	5.21 ± 0.01 ^bC^	4.07 ± 0.03 ^cC^	5.78 ± 0.15 ^aC^	1.64 ± 0.03 ^bC^	1.60 ± 0.31 ^bC^	1.88 ± 0.02 ^aB^
	PL 2	5.40 ± 0.10 ^bC^	3.93 ± 0.06 ^cD^	6.13 ± 0.15 ^aB^	1.65 ± 0.02 ^bC^	1.51 ± 0.03 ^cC^	1.98 ± 0.02 ^aA^
	PL 3	6.34 ± 0.06 ^aB^	3.78 ± 0.15 ^cD^	5.11 ± 0.15 ^bD^	1.88 ± 0.03 ^aA^	1.87 ± 0.02 ^aA^	1.64 ± 0.03 ^bC^
Scenario 2	PL 4	3.93 ± 0.12 ^cD^	5.71 ± 0.1 ^aA^	5.15 ± 0.22 ^bD^	1.69 ± 0.01 ^bC^	1.90 ± 0.01 ^aA^	1.61 ± 0.0.5 ^bC^
	PL 5	6.69 ± 0.1 ^aA^	5.87 ± 0.09 ^bB^	7.09 ± 0.05 ^aA^	1.74 ± 0.01 ^bB^	1.7 ± 0.03 ^bB^	1.87 ± 0.01 ^aB^
	PL 6	6.20 ± 0.03 ^bB^	5.91 ± 0.04 ^cA^	6.85 ± 0.03 ^aA^	1.79 ± 0.02 ^aB^	1.72± 0.02 ^aB^	1.60 ± 0.03 ^bC^

^1^ PL = processing line in the dairy plant. CFU = colony forming unit. Lowercase superscripts within the same row not sharing a common lowercase superscript for each season separately for floor swab samples and air samples are significantly different (*p* < 0.05). Uppercase superscripts within the same column not sharing a common uppercase superscript for each season are significantly different (*p* < 0.05).

**Table 2:** Top twenty predominant species isolated from floor swabs and air samples, listed by prevalence from highest to lowest.

Floor Swab Samples	Air Samples
*Klebsiella oxytoca*	*Micrococcus luteus*
*Shewanella putrefaciens*	*Staphylococcus pasteuri*
*Serratia liquefaciens*	*Pseudomonas fulva*
*Pseudomonas koreensis*	*Peribacillus simplex*
*Pseudomonas veronii*	*Bacillus infantis*
*Pseudomonas fragi*	*Staphylococcus epidermidis*
*Shewanella oneidensis*	*Bacillus thuringiensis*
*Serratia marcescens*	*Bacillus velezensis*
*Leuconostoc mesenteroides*	*Paenibacillus glucanolyticus*
*Pseudomonas gessardii*	*Pseudomonas synxantha*
*Exiguobacterium mexicanum*	*Staphylococcus hominis*
*Pseudomonas gessardii*	*Acinetobacter lwoffii*
*Lelliottia amnigena*	*Staphylococcus warneri*
*Pseudomonas synxantha*	*Staphylococcus saprophyticus*
*Citrobacter gillenii*	*Lysinibacillus fusiformis*
*Serratia proteamaculans*	*Enterococcus casseliflavus*
*Ewingella americana*	*Bacillus mycoides*
*Leuconostoc pseudomesenteroides*	*Stenotrophomonas maltophilia*
*Raoultella ornithinolytica*	*Niallia circulans*
*Pseudomonas fluorescens*	*Arthrobacter parietis*

**Table 3:** Distinct genera present in swab and air samples across two scenarios.

	Genera in Swab	Genera in Air
Scenario 1	*Aeromonas* *Citrobacter* *Enterobacter*	*Acinetobacter* *Aspergillus* *Corynebacterium* *Cytobacillus* *Enterococcus* *Kocuria* *Lysinibacillus* *Metabacillus* *Niallia* *Penicillium* *Peribacillus* *Pseudescherichia* *Stenotrophomonas*
Scenario 2	*Ewingella* *Exiguobacterium* *Hafnei* *Raoultella*	No distinct genera found in Scenario 2 of air samples, all genera identified were similar to Scenario 1 air samples.

## Data Availability

The original contributions presented in this study are included in the article. Further inquiries can be directed to the corresponding author.

## References

[1] Almeida G., Magalhães R., Carneiro L., Santos I., Silva J., Ferreira V., Hogg T., Teixeira P. (2013). Foci of contamination of *Listeria monocytogenes* in different cheese processing plants. Int. J. Food Microbiol..

[2] Carpentier B., Cerf O. (2011). Persistence of Listeria monocytogenes in food industry equipment and premises. Int. J. Food Microbiol..

[3] Ferreira V., Wiedmann M., Teixeira P., Stasiewicz M. (2014). Listeria monocytogenes persistence in food-associated environments: Epidemiology, strain characteristics, and implications for public health. J. Food Prot..

[4] Cortes B.W., Naditz A.L., Anast J.M., Schmitz-Esser S. (2020). Transcriptome sequencing of Listeria monocytogenes reveals major gene expression changes in response to lactic acid stress exposure but a less pronounced response to oxidative stress. Front. Microbiol..

[5] Silva A., Silva V., Gomes J.P., Coelho A., Batista R., Saraiva C., Esteves A., Martins Â., Contente D., Diaz-Formoso L. (2024). Listeria monocytogenes from food products and food associated environments: Antimicrobial resistance, genetic clustering and biofilm Insights. Antibiotics.

[6] CDC (2024). Listeria Outbreak Linked to Meats Sliced at Delis. https://www.cdc.gov/listeria/outbreaks/delimeats-7-24/index.html.

[7] Vidovic S., Paturi G., Gupta S., Fletcher G.C. (2024). Lifestyle of Listeria monocytogenes and food safety: Emerging listericidal technologies in the food industry. Crit. Rev. Food Sci. Nutr..

[8] Anast J.M., Bobik T.A., Schmitz-Esser S. (2020). The Cobalamin-dependent gene cluster of Listeria monocytogenes: Implications for virulence, stress response, and food safety. Front. Microbiol..

[9] Unrath N., McCabe E., Macori G., Fanning S. (2021). Application of whole genome sequencing to aid in deciphering the persistence potential of Listeria monocytogenes in food production environments. Microorganisms.

[10] Osek J., Lachtara B., Wieczorek K. (2022). Listeria monocytogenes–how this pathogen survives in food-production environments?. Front. Microbiol..

[11] Taylor A.J., Stasiewicz M.J. (2019). Persistent and sporadic Listeria monocytogenes strains do not differ when growing at 37 C, in planktonic state, under different food associated stresses or energy sources. BMC Microbiol..

[12] John J., Joy W.-C., Jovana K. (2020). Prevalence of Listeria spp. in produce handling and processing facilities in the Pacific Northwest. Food Microbiol..

[13] Tuytschaever T., Raes K., Sampers I. (2024). Environmental monitoring of a freezing tunnel and its close surroundings in the potato processing industry: Insights into Listeria monocytogenes contamination, cleaning and disinfection efficacy, and transmission risks. Food Control.

[14] Gil M.I., Truchado P., Tudela J.A., Allende A. (2024). Environmental monitoring of three fresh-cut processing facilities reveals harborage sites for Listeria monocytogenes. Food Control.

[15] Dzieciol M., Schornsteiner E., Muhterem-Uyar M., Stessl B., Wagner M., Schmitz-Esser S. (2016). Bacterial diversity of floor drain biofilms and drain waters in a Listeria monocytogenes contaminated food processing environment. Int. J. Food Microbiol..

[16] Wiktorczyk-Kapischke N., Skowron K., Grudlewska-Buda K., Wałecka-Zacharska E., Korkus J., Gospodarek-Komkowska E. (2021). Adaptive response of Listeria monocytogenes to the stress factors in the food processing environment. Front. Microbiol..

[17] Heir E., Møretrø T., Simensen A., Langsrud S. (2018). Listeria monocytogenes strains show large variations in competitive growth in mixed culture biofilms and suspensions with bacteria from food processing environments. Int. J. Food Microbiol..

[18] Rolon M.L., Voloshchuk O., Bartlett K.V., LaBorde L.F., Kovac J. (2024). Multi-species biofilms of environmental microbiota isolated from fruit packing facilities promoted tolerance of Listeria monocytogenes to benzalkonium chloride. Biofilm.

[19] Bremer P.J., Monk I., Osborne C.M. (2001). Survival of Listeria monocytogenes attached to stainless steel surfaces in the presence or absence of *Flavobacterium* spp.. J. Food Prot..

[20] Puga C.H., Dahdouh E., SanJose C., Orgaz B. (2018). Listeria monocytogenes colonizes Pseudomonas fluorescens biofilms and induces matrix over-production. Front. Microbiol..

[21] Fox E.M., Solomon K., Moore J.E., Wall P.G., Fanning S. (2014). Phylogenetic profiles of in-house microflora in drains at a food production facility: Comparison and biocontrol implications of Listeria-positive and-negative bacterial populations. Appl. Environ. Microbiol..

[22] Elias S., Banin E. (2012). Multi-species biofilms: Living with friendly neighbors. FEMS Microbiol. Rev..

[23] Wu W.-J., Park S.-M., Ahn B.-Y. (2013). Isolation and characterization of an antimicrobial substance from Bacillus subtilis BY08 antagonistic to Bacillus cereus and Listeria monocytogenes. Food Sci. Biotechnol..

[24] Stellato G., De Filippis F., La Storia A., Ercolini D. (2015). Coexistence of lactic acid bacteria and potential spoilage microbiota in a dairy processing environment. Appl. Environ. Microbiol..

[25] Schirmer B., Heir E., Møretrø T., Skaar I., Langsrud S. (2013). Microbial background flora in small-scale cheese production facilities does not inhibit growth and surface attachment of Listeria monocytogenes. J. Dairy Sci..

[26] Chowdhury B. (2023). Control of Persistent Environmental Listeria Monocytogenes Using Phenotypic and Genomic Approaches. Electronic Thesis and Dissertations.

[27] Stobnicka-Kupiec A., Gołofit-Szymczak M., Górny R. (2019). Microbial contamination level and microbial diversity of occupational environment in commercial and traditional dairy plants. Ann. Agric. Environ. Med..

[28] Vackova M., Smetana J., Chlibek R., Bostikova V., Splino M. (2011). Microbial air load at the transplant intensive care unit. Mil. Med. Sci. Lett..

[29] Wattal C., Oberoi J., Goel N., Raveendran R., Khanna S. (2017). Matrix-assisted laser desorption ionization time of flight mass spectrometry (MALDI-TOF MS) for rapid identification of micro-organisms in the routine clinical microbiology laboratory. Eur. J. Clin. Microbiol. Infect. Dis..

[30] Singh N., Anand S., Kraus B., Sutariya S. (2021). Evaluating the recovery potential of injured cells of Listeria innocua under product temperature-abuse conditions and passage through simulated gastrointestinal fluids. J. Dairy Sci..

[31] Verma P., Anand S. (2020). Antimicrobial activity as a potential factor influencing the predominance of Bacillus subtilis within the constitutive microflora of a whey reverse osmosis membrane biofilm. J. Dairy Sci..

[32] Choyam S., Lokesh D., Kempaiah B.B., Kammara R. (2015). Assessing the antimicrobial activities of Ocins. Front. Microbiol..

[33] de Farias F.M., Soria M.C., O’Connor P.M., Huang X., Buttimer C., Kamilari E., Deliephan A., Hill D., Fursenko O., Wiese J. (2025). Leuconostoc lactis strain APC 3969 produces a new variant of cyclic bacteriocin leucocyclicin Q and displays potent anti-Clostridium perfringens activity. Sci. Rep..

[34] Sermkaew N., Atipairin A., Krobthong S., Aonbangkhen C., Yingchutrakul Y., Uchiyama J., Songnaka N. (2024). Unveiling a New Antimicrobial Peptide with Efficacy against P. aeruginosa and K. pneumoniae from Mangrove-Derived Paenibacillus thiaminolyticus NNS5-6 and Genomic Analysis. Antibiotics.

[35] Liu N.T., Lefcourt A.M., Nou X., Shelton D.R., Zhang G., Lo Y.M. (2013). Native microflora in fresh-cut produce processing plants and their potentials for biofilm formation. J. Food Prot..

[36] Fagerlund A., Langsrud S., Møretrø T. (2021). Microbial diversity and ecology of biofilms in food industry environments associated with Listeria monocytogenes persistence. Curr. Opin. Food Sci..

[37] Takenaka S., Tonoki T., Taira K., Murakami S., Aoki K. (2007). Adaptation of Pseudomonas sp. strain 7-6 to quaternary ammonium compounds and their degradation via dual pathways. Appl. Environ. Microbiol..

[38] Gunduz G.T., Tuncel G. (2006). Biofilm formation in an ice cream plant. Antonie Van Leeuwenhoek.

[39] Rolon M.L., Chandross-Cohen T., Kaylegian K.E., Roberts R.F., Kovac J. (2024). Context matters: Environmental microbiota from ice cream processing facilities affected the inhibitory performance of two lactic acid bacteria strains against Listeria monocytogenes. Microbiol. Spectr..

[40] Didienne R., Defargues C., Callon C., Meylheuc T., Hulin S., Montel M.-C. (2012). Characteristics of microbial biofilm on wooden vats (‘gerles’) in PDO Salers cheese. Int. J. Food Microbiol..

[41] Tang X., Flint S., Brooks J., Bennett R. (2009). Factors affecting the attachment of micro-organisms isolated from ultrafiltration and reverse osmosis membranes in dairy processing plants. J. Appl. Microbiol..

[42] Calasso M., Ercolini D., Mancini L., Stellato G., Minervini F., Di Cagno R., De Angelis M., Gobbetti M. (2016). Relationships among house, rind and core microbiotas during manufacture of traditional Italian cheeses at the same dairy plant. Food Microbiol..

[43] Oliveira M., Tiwari B.K., Duffy G. (2020). Emerging technologies for aerial decontamination of food storage environments to eliminate microbial cross-contamination. Foods.

[44] Salustiano V.C., Andrade N.J., Brandão S.C.C., Azeredo R.M.C., Lima S.A.K. (2003). Microbiological air quality of processing areas in a dairy plant as evaluated by the sedimentation technique and a one-stage air sampler. Braz. J. Microbiol..

[45] Quigley L., O’Sullivan O., Beresford T.P., Ross R.P., Fitzgerald G.F., Cotter P.D. (2011). Molecular approaches to analysing the microbial composition of raw milk and raw milk cheese. Int. J. Food Microbiol..

[46] Ding Y., Zhou F., Zhou R., Wang Q., Pan S., Wang W. (2025). Microbial Level and Microbiota Change of Laver in Dried Laver Processing Line During Production Seasons. Foods.

[47] Gu G., Ottesen A., Bolten S., Wang L., Luo Y., Rideout S., Lyu S., Nou X. (2019). Impact of routine sanitation on the microbiomes in a fresh produce processing facility. Int. J. Food Microbiol..

[48] Rolon M.L., Tan X., Chung T., Gonzalez-Escalona N., Chen Y., Macarisin D., LaBorde L.F., Kovac J. (2023). The composition of environmental microbiota in three tree fruit packing facilities changed over seasons and contained taxa indicative of L. monocytogenes contamination. Microbiome.

[49] Pathak A.K., Verma K.S. (2013). Assessment of airborne bacteria of milk processing unit complex associated environment. Int. J. Environ. Health Eng..

[50] Tan X., Chung T., Chen Y., Macarisin D., LaBorde L., Kovac J. (2019). The occurrence of Listeria monocytogenes is associated with built environment microbiota in three tree fruit processing facilities. Microbiome.

[51] Liu Y., Zhang H., Wu C., Deng W., Wang D., Zhao G., Song J., Jiang Y. (2016). Molecular analysis of dominant species in Listeria monocytogenes-positive biofilms in the drains of food processing facilities. Appl. Microbiol. Biotechnol..

[52] Oxaran V., Dittmann K.K., Lee S.H., Chaul L.T., Fernandes de Oliveira C.A., Corassin C.H., Alves V.F., De Martinis E.C.P., Gram L. (2018). Behavior of foodborne pathogens Listeria monocytogenes and Staphylococcus aureus in mixed-species biofilms exposed to biocides. Appl. Environ. Microbiol..

[53] Møretrø T., Langsrud S. (2017). Residential bacteria on surfaces in the food industry and their implications for food safety and quality. Compr. Rev. Food Sci. Food Saf..

[54] Xu Z.S., Ju T., Yang X., Gänzle M. (2023). A meta-analysis of bacterial communities in food processing facilities: Driving forces for assembly of core and accessory microbiomes across different food commodities. Microorganisms.

[55] Dailey R.C., Martin K.G., Smiley R.D. (2014). The effects of competition from non-pathogenic foodborne bacteria during the selective enrichment of Listeria monocytogenes using buffered Listeria enrichment broth. Food Microbiol..

[56] Mellefont L., McMeekin T., Ross T. (2008). Effect of relative inoculum concentration on Listeria monocytogenes growth in co-culture. Int. J. Food Microbiol..

[57] Zhao T., Doyle M.P., Zhao P. (2004). Control of Listeria monocytogenes in a biofilm by competitive-exclusion microorganisms. Appl. Environ. Microbiol..

[58] Alonso V.P.P., Harada A.M.M., Kabuki D.Y. (2020). Competitive and/or cooperative interactions of listeria monocytogenes with Bacillus cereus in dual-species biofilm formation. Front. Microbiol..

[59] Li J., Xie S., Ahmed S., Wang F., Gu Y., Zhang C., Chai X., Wu Y., Cai J., Cheng G. (2017). Antimicrobial activity and resistance: Influencing factors. Front. Pharmacol..

[60] Hajdu S., Holinka J., Reichmann S., Hirschl A.M., Graninger W., Presterl E. (2010). Increased temperature enhances the antimicrobial effects of daptomycin, vancomycin, tigecycline, fosfomycin, and cefamandole on staphylococcal biofilms. Antimicrob. Agents Chemother..

[61] Gomes F., Teixeira P., Ceri H., Oliveira R. (2012). Evaluation of antimicrobial activity of certain combinations of antibiotics against in vitroStaphylococcus epidermidis biofilms. Indian J. Med. Res..

[62] Palacios-Rodriguez A.P., Espinoza-Culupú A., Durán Y., Sánchez-Rojas T. (2024). Antimicrobial activity of Bacillus amyloliquefaciens BS4 against Gram-negative pathogenic Bacteria. Antibiotics.

